# Perceptions about COVID-19 vaccine among healthcare workers in Rwanda: A mixed-methods study

**DOI:** 10.4102/jphia.v16i1.668

**Published:** 2025-04-08

**Authors:** Erigene Rutayisire, François X. Ndayambaje, Sembuche Senga, Raji Tajudeen, Darius Uzabakiriho, Solange Nikwigize, Marie F. Muremba, Eric Remera, Tonny Muwonge, Leah Mbabazi, Rodgers R. Ayebare, Francis Kakooza, Tamrat Shaweno, Nebiyu Dereje, Elizabeth Gonese, Mosoka P. Fallah, Ayman Ahmed, Jean Claude S. Ngabonziza

**Affiliations:** 1 College of Medicine and Health Sciences, University of Rwanda, Kigali, Rwanda; 2 African Forum for Research and Education in Health (AFREhealth), Kumasi, Ghana; 3 Africa Centers for Disease Control and Prevention, Dar es Salaam, Tanzania; 4 Africa Centers for Disease Control and Prevention, Addis Ababa, Ethiopia; 5 Division of Research Innovation and Data Science, Rwanda Biomedical Center, Kigali, Rwanda; 6 Infectious Diseases Institute, Makerere University, Kampala, Uganda; 7 Africa Centers for Disease Control and Prevention, Harare, Zimbabwe; 8 Africa Centers for Disease Control and Prevention, Accra, Ghana; 9 Institutes of Endemic Diseases, University of Khartoum, Khartoum, Sudan

**Keywords:** COVID-19, Rwanda, healthcare workers, perception, COVID-19 vaccine, mixed methods

## Abstract

**Background:**

Healthcare workers (HCWs) are crucial for coronavirus disease 2019 (COVID-19) vaccination programmes, but their perceptions of the vaccine, particularly in low-income countries, are underexplored. This study investigated HCWs perceptions of the COVID-19 vaccination in Rwanda.

**Aim:**

This study aimed to understand HCWs’ perceptions of the COVID-19 vaccine in Rwanda.

**Setting:**

A convergent mixed-methods study was conducted in 45 purposively selected health facilities in Rwanda.

**Methods:**

A sample of 230 HCWs was purposively calculated to include 45 health facilities from both rural and urban districts across Rwanda to participate in this study. Healthcare workers were selected conveniently ensuring representation of the different cadres. Furthermore, one participant per facility underwent an in-depth interview. Data were analysed using STATA 17 (quantitative) and Dedoose (qualitative) software. Descriptive analysis was applied and findings presented frequencies and graphical representations. Inductive thematic analysis was performed to identify key themes in the qualitative data.

**Results:**

Most participants were female, 183 (89%), and median age was 39 years. Most were nurses and/or midwives, 98 (42.6%) and all were fully vaccinated. A total of 59 (25.7%) HCWs had little or no confidence in answering patients’ questions about COVID-19 and the vaccine. Despite this, 91.3% would recommend Ministry or World Health Organization (WHO)-approved vaccines and had a positive overall perception about COVID-19 vaccine.

**Conclusion:**

The positive perception of the COVID-19 vaccine among Rwandan HCWs aligns with the country’s successful vaccination programme. This potentially reflects effective national strategies. Further research into Rwanda’s COVID-19 response is however, warranted.

**Contribution:**

This study reveals discrepancies in HCWs vaccine confidence in Rwanda, highlighting the need for targeted educational interventions to strengthen national COVID-19 response efforts.

## Introduction

The coronavirus disease 2019 (COVID-19) pandemic emerged and rapidly spread through the world in early 2020. In a swift global response, efficacious vaccines were developed and distributed worldwide in late 2020 to control the pandemic.^1^ The burden of COVID-19 in Rwanda is characterised by 133 518 cases and 1468 deaths of COVID-19 reported by the World Health Organization (WHO) as of 13 January 2024. A total of 27.3 million vaccine doses have been administered, and 84% of the eligible population has completed the primary vaccine schedule.^2^ Rwanda received the first batch of 1000 doses of COVID-19 vaccines from Moderna in mid-February 2021. Furthermore, on 03 March 2021, Rwanda received 240 000 doses of the AstraZeneca-Oxford COVID-19 vaccine, followed by the first 102 960 doses of the Pfizer vaccine in Africa through the COVAX initiative.^3^

Healthcare workers (HCWs) are at the frontline of the response to health emergencies and pandemics such as the COVID-19 pandemic. They commonly care for patients and control the virus’s spread.^4^ In addition to their natural exposure to risk factors as part of the community and because of the nature of their work and the direct contact with patients infected with different pathogens, frontline HCWs are at high risk of acquiring a wide range of infections, including COVID-19.^5,6^ Because of the high risk of infection and their crucial role in implementing disease prevention and control measures, including immunisation programmes, HCWs were among the first groups to receive the COVID-19 vaccine in Rwanda.^3^ Despite having early access to vaccines and facing both national regulations and institutional mandates for HCW vaccination, the readiness of HCWs to receive the vaccine themselves or to recommend it to their patients remains unclear.

Nevertheless, HCWs play a critical role in promoting health practices in the community, including vaccine acceptance. They are often a trusted source of information for patients and the public.^7^ Therefore, their perceptions and beliefs may influence the message they share with the patients and community. Studies have shown that HCWs who are vaccinated themselves are more likely to promote and advocate for vaccine uptake among their patients and communities.^8,9^

However, there is limited evidence on the perceptions of COVID-19 vaccines among HCWs in low- and middle-income countries (LMICs), including Rwanda. A study done in Ethiopia found that about 40% of HCWs had a negative perception of the COVID-19 vaccine; similarly, this was 34 % in Singapore.^10,11^

As HCWs’ perceptions about COVID-19 vaccines play a key influential role in the vaccine’s acceptability and uptake among their communities, it is crucial to understand their perceptions and underlying beliefs to implement vaccination programmes successfully. Therefore, in this study, we explore HCWs’ perceptions towards COVID-19 vaccination in Rwanda.

The overall objective of this study was to explore the HCW’s perceptions towards COVID-19 vaccines. Specifically, HCWs ability to communicate with patients and trusted sources about COVID-19 vaccine information. Our study was guided by the following research questions:

- *What are the trusted sources of COVID-19 vaccine information?*
- *What is the ability of the HCWs to communicate with patients about COVID-19 vaccine?*
- *Are HCWs willing to recommend COVID-19 vaccine to others?*

## Research methods and design

### Study design and setting

This was a cross-sectional convergent mixed-methods study involving both primary quantitative and qualitative data collection. The study was conducted in Rwanda, a low-income country with a population of 13 246 394 as of August 2022.^12^ Rwanda’s healthcare system is led by the Ministry of Health and includes various institutions managing policies and resources. The country has a network of public and private health facilities providing various healthcare services.^13^

### Study population and sampling strategy

A sample of 230 HCWs was purposively estimated. Healthcare workers were sampled from 45 health facilities from both rural and urban districts across Rwanda to participate in this study. Healthcare workers were selected conveniently ensuring adequate representation of the different cadres from various geographic locations, both rural and urban, as well as different types of facilities. The inclusion criteria required participants to be HCWs aged 18 years and older who provided informed consent. For the qualitative aspect of the study, one key informant, an HCW actively involved in managing COVID-19 cases during the pandemic, was selected from each of the 45 health facilities, resulting in a total of 45 key informants.

### Data collection

The data collection tools were developed following the ‘WHO – Evaluation of COVID-19 Vaccine Effectiveness Guide (2021)’^14^ and validated through a pilot session to ensure their relevance to the research questions. A structured questionnaire based on the behavioural and social drivers model^15^ was used to collect data on demographics including gender (male or female), age in complete years, number of vaccination doses received, vaccination experiences, perceptions and information sources (Appendix 1). It explored the likelihood of recommending the vaccine, confidence in addressing patient queries, barriers to vaccination and reasons for hesitancy or refusal. Furthermore, it asked about trust levels in various information sources about COVID-19 and vaccinations. Within each health facility, five HCWs who had direct interactions with COVID-19 patients were randomly selected for participation in the quantitative phase using a simple random sampling method. The data collection process was meticulous, ensuring the reliability and validity of the quantitative data through data collector training and support supervision along with continuous data audits and cleaning. Data were collected within 3 months, from 08 May 2023 to 02 July 2023 and entered into REDcap, an online data management platform, promptly. Trained researchers conducted qualitative interviews to ensure consistency and rigorous data collection.

In-depth interviews were conducted with 45 key informants (one from each health facility selected) to explore their perspectives in more detail. Interviews were audio-recorded and transcribed, with one exception where detailed notes were used. The interviews allowed flexibility in exploring the key informants’ perspectives and perceptions towards COVID-19 vaccination, ensuring a rich and comprehensive qualitative dataset. The interviews were conducted within the key informants’ places of work to create a comfortable and familiar setting, promoting open and honest discussions. Almost all interviews were conducted in the local ‘Kinyarwanda’ language, and only one was conducted in English. Transcription was done in Kinyarwanda and then back-translated to English by an independent consultant to ensure reliability.

### Data analysis

The data analysis was conducted using STATA version 17 (StataCorp, College Station, Texas, United States [US]) for the quantitative portion, where descriptive statistics such as frequencies and percentages were employed to summarise the findings; graphical representations were also created to enhance the understanding of the data. For the qualitative analysis, an external consultant was engaged to code and analyse the data using Dedoose software (University of California, Los Angeles, Unites States [US]), which helped reduce bias in the process. An inductive thematic analysis was then performed to identify key themes within the coded data.

### Ethical considerations

Ethical approval to conduct this study was obtained from the Rwanda National Research Ethics Committee (RNEC) (IRB 00001497 of IORG0001100; Ref No.100/ RNEC/2023). Written informed consent was obtained from all participants after providing them with information about the study. Data were anonymised to ensure participant confidentiality.

## Results

Of the 230 HCWs, 183 (79.6%) were female and 47 (20.4%) were male. The majority of HCWs, 93 (40.4%), were aged between 30 years and 39 years, and the median (interquartile range [IQR]) age was 39 (33–44) years. The study enrolled 98 (42.6%) nurses and midwives. Participants from health centres located in urban areas were 139 (60.4%), whereas 21 (9.2%) and 70 (30.4%) were in semi-urban and rural areas, respectively. All participants in this study were fully vaccinated, and most had received a booster, with only four (1.7%) having not received the booster dose (Table 1).

**TABLE 1:** Demographic characteristics of respondents and vaccination coverage.

Variables	Frequency (*n*)	Percentage	Mean	IQR
**Gender**			-	-
Female	183	79.6	-	-
Male	47	20.4	-	-
**Age (years)**			39	33–44
21–29	32	13.9	-	-
30–39	93	40.4	-	-
40–49	75	32.6	-	-
50–62	30	13.0	-	-
**Health facility location**			-	-
Urban	139	60.4	-	-
Semi-urban	21	9.1	-	-
Rural	70	30.4	-	-
**COVID-19 vaccine status**			-	-
Didn’t receive a booster dose	4	1.7	-	-
Received a booster dose	226	98.3	-	-
**Healthcare worker cadre**			-	-
Physicians	19	8.3	-	-
Nursing and midwifery	98	42.6	-	-
Pharmaceutical personnel	17	7.4	-	-
Laboratory health workers	33	14.4	-	-
Community and public health workers	22	9.6	-	-
Other health workers	41	17.8	-	-
**Preferable place to get COVID-19 vaccine**			-	-
Health centre	209	90.9	-	-
Pharmacy	3	1.3	-	-
Community centre/Health post	13	5.7	-	-
Any other location designated by MoH	21	9.1	-	-

MoH, Ministry of Health; COVID-19, coronavirus disease 2019; IQR, interquartile range.

Most HCWs, 209 (90.9%), were comfortable receiving the vaccine at the health centre. In comparison, 21 (9.1%) healthcare providers were comfortable receiving the vaccine from any other location designated by the Ministry of Health (MoH) (Table 1). Participants also shared that the community preferred receiving the vaccines from health facilities, as one midwife described in an in-depth interview:

‘The vaccines were found at the health centres or vaccination sites set up by MoH/RBC, but most people preferred to come to the health centre; of course, they are used to coming here.’ (HCW02, female, midwife)

**FIGURE 1:**
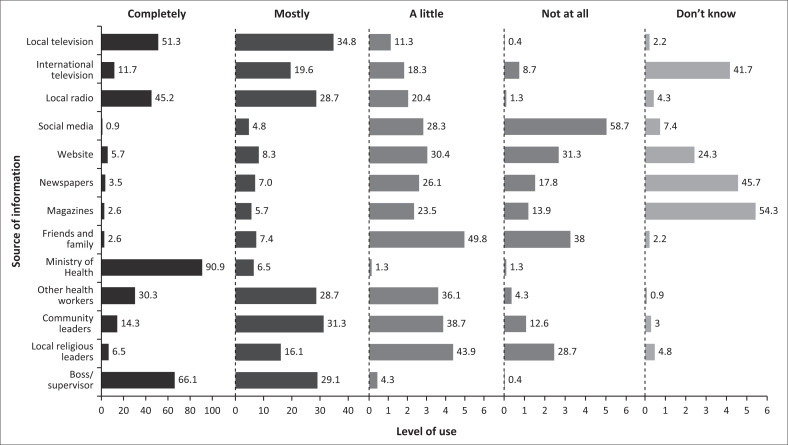
Trust in information sources about coronavirus disease 2019 and vaccination.

During the COVID-19 pandemic, different communication platforms were used to disseminate related information. In this study, 209 (90.9%) HCWs said they had complete trust in the information about COVID-19 and vaccination provided by the Rwanda MoH, and 152 (66.1%) HCWs had complete confidence in the information provided by their supervisors. However, most HCWs, 135 (58.7%), did not trust information about COVID-19 on social media (Figure 1). The key informants reiterated the doubt in social media information and strong trust in government information sources as expressed by this participant:

‘The Vaccine came before we were prepared for it. We doubted at first time because of wrong information that was shared on social media and networking sites, newspapers and on the radio where you heard about vaccines and the people who refused to have them but when we heard some news on radio and television, we knew that our leaders would not give something wrong to their people.’ (HCW34, female, nurse)

According to 218 (94.8%) HCWs, COVID-19 vaccination services were easily accessible (Table 2). Key informants emphasised that they were accessible because there was access to transport modes such as motorcycles and that the requirements to attain vaccination were easy:

‘We have had no problem getting the COVID-19 vaccine. First, at the District Hospital, they are available; if they are not available there, they give us the schedule, and we go to the command post so that we would never miss the vaccines whenever we need them. We never had that case. Also, the Ministry of Health has created an easy way to get these vaccines because it provides us with transportation.’ (HCW14, male, nurse)

**TABLE 2:** Healthcare worker’s willingness to recommend coronavirus disease 2019 vaccination to eligible individuals in their community.

Indicators	Frequency (*n*)	Percentage
**Access to COVID-19 vaccination services**		
Moderately easy	9	3.9
Not at all easy	3	1.3
Very easy	218	94.8
**Recommend COVID-19 vaccinations to the community member**		
Definitely recommend	216	93.9
I do not know	2	0.9
Probably not recommend	1	0.4
Probably recommend	11	4.8
**Recommend available COVID-19 boosters to eligible individuals**		
I would not recommend booster vaccine dosing	2	0.9
I would recommend booster vaccine doses recommended by the WHO	87	37.8
I would recommend some booster vaccine schedules and not others	18	7.8
I would recommend the booster vaccine doses recommended by the MoH in my country	123	53.5
**Confident in answering patient questions about COVID-19 and vaccine**		
A little confident	54	23.5
Moderately confident	84	36.5
Not at all confident	5	2.2
Very confident	87	37.8

COVID-19, coronavirus disease 2019; WHO, World Health Organization.

A total of 206 (89.6%) HCWs believed that being vaccinated against COVID-19 reduces the risk of a person getting sick or dying, and 216 (93.9%) HCWs agreed that they would recommend COVID-19 vaccination to community members. A total of 87 (37.8%) HCWs said that they would recommend booster vaccine doses recommended by the WHO, and 123 (53.5%) HCWs would recommend those recommended by the Rwanda MoH (see Table 2). Moreover, the key informants emphasised their willingness to encourage community members to take on the vaccines:

‘Our role is to show them/patients the benefits of the vaccine and usually talk about it at health centres and hospitals so that those people who have not taken vaccine could take it.’ (HCW31, female, nurse)

While 54 (23.5%) HCWs reported little confidence in answering patient questions about COVID-19 and vaccines, 87 (37.8%) HCWs reported that they would be very confident in doing so (see Table 2). However, the in-depth interviews with key informants revealed that because of limited knowledge of the vaccines, they were unsure how to respond to patient queries about the COVID-19 vaccines, as expressed by one nurse:

‘As the vaccine caused concern, people were worried because it was the first vaccine that entered the population, then there was no one else, let’s say a doctor, we did not know what side effects were, we had no experience with it, so when someone came to you and told you that he had a problem, you tried to explain to him because it was in the system, you go If you look at Google, there are times when you see something wrong because there is no reliable information about it, there are no experts.’ (HCW43, female, nurse)

## Discussion

This study explored HCWs’ perceptions of COVID-19 vaccination in Rwanda. The findings offer valuable insights into their level of trust in information sources, confidence in vaccines and willingness to recommend vaccination to the community. They align with broader research on HCW vaccine hesitancy.

The results demonstrated a high trust in the MoH and a preference for health facilities for vaccination. The majority of HCWs expressed trust in information from the Rwanda MoH and preferred receiving vaccines at health facilities. This contradicts the findings from other sub-Saharan African countries where low trust in the MoH and Government was reported.^16,17^ High trust in government information sources on COVID-19 has been chiefly consistent with findings from high-income countries such as the United States.^18^ Trust in formal information sources about COVID-19 has previously been found to influence vaccine uptake.^19^ Therefore, the trust reported by the participants could explain the 100% vaccination coverage in this group.

This study also highlighted concerns about misinformation and lack of confidence in COVID-19 vaccines. This is consistent with global trends reported in Germany,^20^ India,^21^ and in a global review,^22^ highlighting the widespread challenge of misinformation and its impact on vaccine hesitancy among HCWs.

Concerning accessibility, the study found that HCWs perceived COVID-19 vaccination services as readily accessible. This differs from the experiences reported in neighbouring Kenya and South Africa, where a study found that less than a quarter of the HCWs reported that accessing vaccination services for themselves was easy.^23^ The reported higher accessibility to vaccination services could be attributed to Rwanda’s strong health governance and policy implementation^24,25^ that was applied to implementing COVID-19 vaccination mandates for HCWs. In addition, the majority (over 90%) expressed willingness to recommend vaccination to community members, demonstrating their commitment to promoting public health, as observed in studies from Ghana^26^ and Jordan.^27^

Limitations in addressing patient questions: Despite their willingness, a significant proportion of HCWs (23%) reported limited confidence in answering patient questions about COVID-19 and vaccines. This echoes concerns raised in Kenya^28^ and Angola,^29^ highlighting the need for targeted training programmes to equip HCWs with the knowledge and skills to address vaccine-related inquiries effectively. Similarly, in Japan, a study revealed that HCWs had low confidence regarding COVID-19 care.^30^ This was also demonstrated in another study where only 39.4% of HCWs showed adequate self-confidence in applying infection control COVID-19 measures.^31^

The findings of this study highlight the importance of addressing misinformation through effective communication strategies that are tailored to the concerns and preferred information channels of HCWs. It is crucial to build trust in COVID-19 vaccines by providing accurate information that emphasises their benefits and addresses specific concerns raised by HCWs, which underscores the necessity of pharmacovigilance programmes. Moreover, equipping HCWs with the knowledge and skills needed to confidently answer patients’ questions about COVID-19 and vaccines can be achieved through targeted training programmes. Future studies should explore the specific reasons for HCWs’ lack of confidence in COVID-19 vaccines within the Rwandan context, considering potential differences across various professional cadres and regions. Researchers should also identify the most effective communication strategies to combat misinformation and build trust among HCWs in Rwanda, taking into account the local media landscape and information consumption habits. Finally, assessing the impact of training programmes on HCWs’ confidence in addressing patient inquiries about COVID-19 and vaccines will be important, including measuring changes in knowledge, attitudes and self-reported confidence. By addressing these issues and building upon existing research, Rwanda’s policymakers and public health officials can develop targeted interventions to improve vaccine confidence and uptake among HCWs and the broader community, ultimately contributing to the success of COVID-19 vaccination efforts and other initiatives aimed at preventing vaccine-preventable diseases.

This study’s limitation is that the focus was restricted solely to HCWs, overlooking the broader spectrum of multisectoral involvement. Consequently, the findings may not reflect a comprehensive explanation for the remarkable success of the vaccination programme in Rwanda.

## Conclusion

Understanding and addressing the perceptions of HCWs regarding the COVID-19 vaccine is crucial for effective vaccination campaigns, public health strategies and overall pandemic control. The overwhelming positive perceptions of the COVID-19 vaccine among Rwandans HCW echo the massive adoption of the COVID-19 vaccine in Rwanda. Such exceptional positive perceptions about COVID-19 vaccines among HCWs warrant further analyses to explore the promoting factors, including the broad spectrum that led to Rwanda’s super successful vaccination programme. We recommend maintaining open and transparent communication about vaccine distribution, safety and effectiveness, especially for new vaccines.

## Appendix 1: Questionnaire

**Questionnaire on COVID-19 vaccine acceptance and uptake among healthcare workers**

**To be completed by the questionnaire administrator:**

**Table:**

Date of Interview (DD/MM/YYYY):	Participant ID (please copy on the top page of each page of the Questionnaire):
Health Facility Name:	

### Instructions to participant

Welcome to this session, we appreciate your willingness to complete this brief survey. This survey aims to understand better access and attitudes to coronavirus disease 2019 (COVID-19) vaccinations among healthcare workers. You are being asked to participate in this survey because of your experience as a healthcare worker (HCW) during the COVID-19 pandemic. This survey will take 10 min or less to complete.

The risks of being involved with this survey are minimal. There are no penalties for not participating in this survey. For example, no employment repercussions will occur to you if you choose not to participate. Participation is entirely voluntary, and at any point, you may decide to discontinue your participation. There are no direct costs or benefits to you for participating in this questionnaire. However, your participation could help us understand how to best target interventions to promote COVID-19 vaccine uptake to keep HCWs safe during this pandemic.

Your participation in this survey will be strictly confidential. None of the information you share with us will be associated with your name. Only staff involved in the analysis and/or review of the questionnaire responses will access the data. Every effort possible will be taken to ensure your privacy, including the deidentification of data. However, we can’t guarantee it. There may be unforeseeable risks that we can’t guarantee 100% privacy. You can end your participation without penalty at any point during this survey process. Thank you for your time.

### May we proceed with the questionnaire?

≤ Yes, we can proceed.

≤ No, I do not want to proceed: *STOP and identify another participant and complete a new questionnaire form*

**Firstly, I would like to know some information about you and your facility.**

**Table:**

**1. How old are you? (In years, select only ONE response)** – *coded as a CATEGORICAL response variable, with categories as below*	
1a. □ 18–29 years 1b. □ 30–39 years 1c. □ 40–49 years	1d. □ 50–59 years 1e. □ 60 years or older 1f. □ I do not wish to respond

**Table:**

**2. What gender do you identify as? (Select only ONE response)** – *coded as a CATEGORICAL response variable, with categories as below*	
2a. □ Female 2b. □ Male	2c. □ Other 3d. □ I do not wish to respond

**Table:**

**3. What job best describes the one you do at this facility? (Select only ONE response)** – *coded as a CATEGORICAL response variable, with categories as below*	
3a. □ Physicians (doctors, clinical officers & dentists) 3b. □ Nurse/midwife 3d. □ Pharmaceutical personnel 3e. □ Laboratory health workers	3.f □ Community support and public health workers 3.g □ Other health workers (specify)

**Table:**

**4. What sector is your health facility in?** (Select only ONE response) – *coded as a CATEGORICAL response variable, with categories as below*
4a. □ Public 4b. □ Private 4c. □ Private not for profit

**Table:**

**5. What is the geographical location of your health facility?** (Select only ONE response) – *coded as a CATEGORICAL response variable, with categories as below*
5a. □ Urban 5b. □ Rural/Peri-urban

Secondly, I would like to ask you some questions about vaccines. Please choose the answer that best captures your belief or understanding.

**Table:**

**6. How much do you agree with this statement: ‘I believe being vaccinated against infectious diseases (i.e. measles, tuberculosis) reduces the risk of a person getting sick or dying’** (select only ONE response) – *coded as a CATEGORICAL response variable, with categories as below*	
6a. □ Strongly disagree 6b. □ Disagree somewhat 6c. □ Neither agree nor disagree 6d. □ Agree somewhat 6e. □ Strongly agree	6f. □ I don’t know 6g. □ I do not wish to respond

**Table:**

**7. How much do you agree with this statement: ‘I believe being vaccinated against COVID-19 reduces the risk of a person getting sick or dying**’ (select only ONE response) – *coded as a CATEGORICAL response variable, with categories as below*	
7a. □ Strongly agree 7b. □ Agree somewhat 7c. □ Neither agree nor disagree 7d. □ Disagree somewhat 7e. □ Strongly disagree	7f. □ I don’t know 7g. □ I do not wish to respond
**8. How many doses have you received of the approved COVID-19 vaccine?** (Select only ONE response) – *coded as a CATEGORICAL response variable, with categories as below*	
8a. □ One dose of a two-dose vaccine — Continue survey 8b. □ One dose of a single dose vaccine — Continue survey 8c. □ Two doses à Continue survey 8d. □ I have received a booster dose — Skip 13 and 14 8e. □ None yet, but I intend to get it — Skip 9 8f. □ None, I do not want to get it — Skip 9 8g. □ I do not know — Skip 9 8h. □ I do not wish to respond — Skip 9	
**9. How easy was it to get vaccination services for yourself, or how easy is it?** (Select only ONE response) – *coded as a CATEGORICAL response variable, with categories as below*	
9a. □ Not at all easy 9b. □ A little easy 9c. □ Moderately easy 9d. □ Very Easy	
**10. Where would you prefer to get a COVID-19 vaccine?** (Prompt the respondent and tick all applicable responses)	
10a. □ Hospital 10b. □ Health centre/clinic 10c. □ Pharmacy 10d. □ Community centre, meeting hall, or place of worship 10e. □ Somewhere else, please specify: __________________________ 10f. □ I don’t want the vaccine	
**11. How likely are you to recommend COVID-19 vaccinations to your community members and patients prioritised by the country to receive the vaccine?** (Select only ONE response) – *coded as a CATEGORICAL response variable, with categories as below*	
11a. □ I Definitely recommend 11b. □ Probably recommend 11c. □ Probably not recommend 11d. □ Definitely not recommend	11e. □ I don’t know 11f. □ I do not wish to respond
**12. As a health worker, would you recommend available COVID-19 vaccines to eligible patients?** (Select only ONE response) – coded as a CATEGORICAL response variable, with categories as below	
12a. □ I would recommend any COVID-19 vaccine that has been authorised/approved by my country 12b. □ I would only recommend COVID-19 vaccines that WHO has recommended 12c. □ I would recommend some COVID-19 vaccines, but not others 12d. □ I would not recommend any COVID-19 vaccines 12e. □ Unsure if I will recommend a COVID-19 vaccine	

**Table:**

**13. How confident are you that you could answer patient questions about COVID-19 vaccines available in the country?** (Select only ONE response) – coded as a CATEGORICAL response variable, with categories as below
13a. □ Not at all confident 13b. □ A little confident 13c. □ Moderately confident 13d. □ Very confident 13e. □ Unsure

**Table:**

**14. You mentioned that you haven’t yet received the vaccine or are not yet fully vaccinated and/or boosted. What are some of the reasons why?** (Select ALL responses that apply) – *coded as individual BINARY response variables*	
14a. □ I don’t know where to go to get a vaccine 14b. □ I don’t have time to get vaccinated 14c. □ I don’t have transportation or am unable to get to the vaccination site 14d. □ I am concerned about the cost of vaccination 14e. □ I am waiting to see how the vaccine affects other people that I know	14f. □ I am worried about the safety of the vaccine, including side effects 14g. □ I recently recovered from COVID-19 14h. □ I don’t want to miss work 14i. □ Another reason (SPECIFY below): 14j. □ I do not wish to respond

**Table:**

**15. What are some of the reasons you would not want to receive an approved COVID-19 vaccine and/or booster?** (OPEN-ENDED question, interviewer to select ALL responses that apply) – *coded as individual BINARY response variables*	
15a. □ I do not feel I am at risk of catching the virus 15b. □ I do not think I am at risk of getting very sick or dying from the virus 15c. □ I am confident there will be other effective treatments soon 15d. □ I do not yet know enough about the vaccine to decide 15e. □ I feel the development and/or authorisation of the vaccine was rushed, and it may not be thoroughly tested 15f. □ Vaccines are against my religious beliefs 15g. □ Vaccines can give you the disease they are designed to protect you against 15h. □ The vaccine availably in my country won’t protect me 15i. □ I want a different vaccine than the one(s) available in my district/locality now	15j. □ People in my community are fearful about being near people who have been vaccinated 15k. □ I am concerned about serious side effects, like blood clots, neurological disorders, or effects on motherhood 15l. □ Other concerns about conspiracies they might have heard about include micro-chips, sterilisation, tracking, etc. 15m. □ I already had COVID-19 and am not worried about being infected again 15n. □ Someone in my family or community does not want me to get the vaccine 15o. □ OTHER (Specify below): 15p. □ This does not apply to me. I am willing to receive the vaccine

**Table:**

**16. What do you think will help in your decision to get vaccinated?** (OPEN-ENDED question, interviewer to select ALL responses that apply)
16a. □ More information on vaccine safety and efficacy 16b. □ Full approval of the vaccine from regulatory authorities 16c. □ Better resources for appointment scheduling 16d □ Closer vaccine administration facilities 16e □ Nothing. Will not get vaccinated 16f. □ Other specify

**Table:**

17. How much do you trust the following information sources and platforms about COVID-19 and vaccinations? (choose ONE response for each source below)					
	Completely	Mostly	A little	Not at all	Don’t know/ do not get information from this source
17a. Local television					
17b. International television channel					
17c. Local radio					
17d. Social media (Twitter, Facebook, WhatsApp, Instagram)					
17e. Websites					
17f. Newspapers					
17g. Magazines					
17h. Friends and family					
17i. Ministry of Health					
15j. Other health workers					
17k. Community leaders					
17l. Local religious leaders					
17m. Boss/Supervisor					

## References

[1] Mathieu E, Ritchie H, Rodés-Guirao L, et al. Coronavirus pandemic (COVID-19). Our world in data [homepage on the Internet]. 2020 [cited 2024 Mar 14]. Available from: https://ourworldindata.org/coronavirus

[2] WHO. COVID-19 Rwanda situation. WHO COVID-19 dashboard [homepage on the Internet]. [cited 2024 Mar 14]. Available from: https://data.who.int/dashboards/covid19/cases?m49=646&n=c

[3] Nsanzabaganwa C, Bigirimana N, Hitimana N, et al. Rwanda COVID-19 vaccination program implementation [homepage on the Internet]. Kigali; 2021 [cited 2024 Mar 14]. Available from: https://rbc.gov.rw/publichealthbulletin/img/RPHB%20Vol.%203,%20issue%201.%20pp7-9.pdf

[4] Hoernke K, Djellouli N, Andrews L, et al. Frontline healthcare workers’ experiences with personal protective equipment during the COVID-19 pandemic in the UK: A rapid qualitative appraisal. BMJ Open. 2021;11:e046199. 10.1136/bmjopen-2020-046199 PMC7818840 33472794

[5] Moyo I, Mavhandu-Mudzusi AH, Haruzivishe C. Frontline healthcare workers’ experiences of providing care during the COVID-19 pandemic at a COVID-19 centre in Bulawayo, Zimbabwe: A phenomenological study. Curationis. 2022;45(1):e1–e11. 10.4102/curationis.v45i1.2292 PMC9257684 35792610

[6] Nguyen LH, Drew DA, Graham MS, et al. Risk of COVID-19 among front-line health-care workers and the general community: A prospective cohort study. Lancet Public Health [serial online]. 2020 [cited 2024 Mar 14];5:e475–e483. Available from: http://www.thelancet.com/article/S246826672030164X/fulltext 32745512 10.1016/S2468-2667(20)30164-X PMC7491202

[7] Niyigena A, Nyirahabimana N, Cubaka V, et al. Knowledge and practices surrounding outbreaks and COVID-19 among community health workers in rural Rwanda: A cross-sectional mixed-methods study. Pan Afr Med J. 2023;45:35. 10.11604/pamj.2023.45.35.37020 37545611 PMC10403765

[8] Alhassan RK, Owusu-Agyei S, Ansah EK, Gyapong M. COVID-19 vaccine uptake among health care workers in Ghana: A case for targeted vaccine deployment campaigns in the global south. Hum Resour Health. 2021;19:1–12. 10.1186/s12960-021-00657-1 34742301 PMC8571849

[9] Biswas N, Mustapha T, Khubchandani J, Price JH. The nature and extent of COVID-19 vaccination hesitancy in healthcare workers. J Community Health. 2021;46:1244–1251. 10.1007/s10900-021-00984-3 33877534 PMC8056370

[10] Adane M, Ademas A, Kloos H. Knowledge, attitudes, and perceptions of COVID-19 vaccine and refusal to receive COVID-19 vaccine among healthcare workers in northeastern Ethiopia. BMC Public Health. 2022;22:1–14. 10.1186/s12889-021-12362-8 35042476 PMC8765812

[11] Lim EH, Fong NP, Pang J. Factors of COVID-19 vaccine perception among transport drivers in singapore: A cross-sectional pilot study. Am J Trop Med Hyg. 2023;108:588. 10.4269/ajtmh.22-0510 36746661 PMC9978561

[12] National Institute of Statistics of Rwanda. Main indicators: 5th Rwanda Population and Housing Census (PHC) [homepage on the Internet]. Kigali: National Institute of Statistics Rwanda; 2023 [cited 2024 Mar 14]. Available from: https://www.statistics.gov.rw/publication/main_indicators_2022

[13] Ministry of Health Rwanda. Health sector annual performance report 2020–2021 [homepage on the Internet]. Kigali; 2022 [cited 2024 Mar 14]. Available from: https://www.moh.gov.rw/index.php?eID=dumpFile&t=f&f=36820&token=2e1aac6615a585b990697f58e2915ee3ee2c6f9a

[14] Patel MK, Bergeri I, Bresee JS, et al. Evaluation of post-introduction COVID-19 vaccine effectiveness: Summary of interim guidance of the World Health Organization. Vaccine. 2021;39:4013. 10.1016/j.vaccine.2021.05.099 34119350 PMC8166525

[15] Brewer NT, Chapman GB, Rothman AJ, Leask J, Kempe A. Increasing vaccination: Putting psychological science into action. Psychol Sci Public Interest. 2017;18:149–207. 10.1177/1529100618760521 29611455

[16] Mohammed R, Nguse TM, Habte BM, Fentie AM, Gebretekle GB. COVID-19 vaccine hesitancy among Ethiopian healthcare workers. PLoS One. 2021;16(12):e0261125. 10.1371/journal.pone.0261125 34919597 PMC8682893

[17] Amuzie CI, Odini F, Kalu KU, et al. Covid-19 vaccine hesitancy among healthcare workers and its socio-demographic determinants in abia state, southeastern nigeria: A cross-sectional study. Pan Afr Med J. 2021;40:10. 10.11604/pamj.2021.40.10.29816 PMC8490164 34650660

[18] Li H, Chen B, Chen Z, et al. Trust in COVID-19 information from different media types and its association with preventive measures adoption in the U.S. J Health Commun. 2023;28:633–647. 10.1080/10810730.2023.2245373 37665096 PMC11929583

[19] Maykrantz SA, Gong T, Petrolino AV, Nobiling BD, Houghton JD. How trust in information sources influences preventative measures compliance during the COVID-19 pandemic. Int J Environ Res Public Health. 2021;18(11):5867. 10.3390/ijerph18115867 34070713 PMC8198292

[20] Holzmann-Littig C, Braunisch MC, Kranke P, et al. Covid-19 vaccination acceptance and hesitancy among healthcare workers in Germany. Vaccines (Basel). 2021;9(7):777. 10.3390/vaccines9070777 34358193 PMC8310090

[21] Jain J, Saurabh S, Kumar P, et al. COVID-19 vaccine hesitancy among medical students in India. Epidemiol Infect. 2021;149:e132. 10.1017/S0950268821001205 34011421 PMC8185413

[22] Khubchandani J, Bustos E, Chowdhury S, Biswas N, Keller T. COVID-19 vaccine refusal among nurses worldwide: Review of trends and predictors. Vaccines (Basel). 2022;10(2):230. 10.3390/vaccines10020230 35214687 PMC8876951

[23] Bon HB, Brouwers SA, Mote J, et al. Measuring behavioral and social drivers of COVID-19 vaccination in health workers in Eastern and Southern Africa. BMC Proc. 2023;17(Suppl 7):14. 10.1186/s12919-023-00262-1 37438751 PMC10337050

[24] Iyer HS, Chukwuma A, Mugunga JC, et al. A comparison of health achievements in Rwanda and Burundi. Health Hum Rights. 2018;20:199. 30008563 PMC6039746

[25] Leslie FM, Ravishankar N, Squires J, Williamson TR, Brinkerhoff D. Rwanda health governance assessment [homepage on the Internet]. 2010 [cited 2024 Mar 14]. Available from: https://www.hfgproject.org/wp-content/uploads/2015/02/Rwanda-Health-Governance-Assessment.pdf

[26] Mohammed AS, Asumah MN, Padhi BK, et al. Predictors of SARS-CoV-2 vaccine uptake among health professionals: A cross-sectional study in Ghana. Vaccines (Basel). 2023;11(1):190. 10.3390/vaccines11010190 36680035 PMC9864896

[27] Lubad MA, Abu-Helalah MA, Alahmad IF, et al. Willingness of healthcare workers to recommend or receive a third COVID-19 vaccine dose: A cross-sectional study from Jordan. Infect Dis Rep. 2023;15:210–221. 10.3390/idr15020022 37102982 PMC10138052

[28] Abdulle HM, Masika MM, Oyugi JO. COVID-19: Knowledge, perception of risk, preparedness and vaccine acceptability among healthcare workers in Kenya. Pan Afr Med J. 2022;41:239. 10.11604/pamj.2022.41.239.33985 35721652 PMC9167448

[29] Arrais M, DIas W, Gama JMR, Brito M. Physicians’ perceptions of their knowledge and the preparedness of health facilities in Angola to diagnose and manage COVID-19. Int Health. 2022;14:103–110. 10.1093/inthealth/ihab017 33845486 PMC8083292

[30] Kadoya Y, Zen K, Wakana N, et al. Knowledge, perception, and level of confidence regarding COVID-19 care among healthcare workers involved in cardiovascular medicine: A web-based cross-sectional survey in Japan. J Cardiol. 2021;77:239. 10.1016/j.jjcc.2020.07.029 32859452 PMC7414383

[31] Elgibaly O, Daef E, Elghazally SA, et al. Knowledge, perception, and confidence of healthcare workers about COVID-19 preventive measures during the first wave of the pandemic: A cross-sectional study from Egypt. Germs. 2021;11(2):179–188. 10.18683/germs.2021.1255 34422690 PMC8373401

